# Prevalence of Electrolyte Abnormality and its Correlation with Clinical Features and Patient Outcomes in Children Admitted to Pediatric Intensive Care Unit of a Resource-Constrained Setting in India

**DOI:** 10.1177/20503121251391990

**Published:** 2025-11-29

**Authors:** Arushi Gupta, Sunil Kasundriya, Shreya Shrivastava, Manju Purohit, Ashish Pathak

**Affiliations:** 1 Department of Pediatrics, Ruxmaniben Deepchand Gardi Medical College, Ujjain, Madhya Pradesh, India; 2 Department of Global Public Health, Health Systems and Policy (HSP): Medicines, Focusing Antibiotics, Karolinska Institutet, Stockholm, Sweden; 3 Department of Pathology, Ruxmaniben Deepchand Gardi Medical College, Ujjain, Madhya Pradesh, India

**Keywords:** Electrolyte imbalance, pediatric intensive care unit, hypocalcemia, hypokalemia, hyponatremia, mortality

## Abstract

**Background:**

Pediatric patients with critical illnesses often exhibit serum electrolyte disturbances, which significantly influence their clinical outcomes.

**Methods:**

This prospective observational study included 534 pediatric patients (age range 1 month to 18 years) admitted to the pediatric intensive care unit of R.D. Gardi Medical College, Ujjain, from January 1, 2023 to April 30, 2024. The objective of the study was to study associations of disturbances in the levels of serum sodium, potassium, calcium, magnesium, and phosphorus with organ system involvement and child mortality, using multiple logistic regression models.

**Results:**

Among the 534 patients, 325 (61%) were boys and 209 (39%) were girls, with a mean ± SD age of 5.17 ± 4.79 years. Most patients presented with fever (71%), fatigue (34%), and malaise (21%). A total of 1057 electrolyte abnormalities were recorded, with hypocalcemia (263 episodes, 25%) being the most common, followed by hypokalemia (192 episodes, 18%) and hyponatremia (172 episodes, 16%). A total of 173 episodes of severe hypocalcemia (S. calcium <6.5 mg/d), 21 episodes of severe hypernatremia (S. sodium >150 mEq/L) and 12 episodes of hypokalemia (S. potassium <2.5 mEq/L). All children with hypocalcemia had neurological symptoms. Hypokalemia was significantly associated with neurological (adjusted odds ratio 2.07), endocrine (adjusted odds ratio 2.26) and cardiovascular system (adjusted odds ratio 10.20) symptoms. Hyponatremia was significantly associated with symptoms of respiratory system (adjusted odds ratio 2.82) and gastrointestinal system (adjusted odds ratio 1.95). Hyperkalemia was significantly associated with symptoms of neurological (adjusted odds ratio 3.84), endocrine (adjusted odds ratio 2.24) and cardiovascular system (adjusted odds ratio 3.53). A total of 34 (6%) deaths were recorded and found to be associated mainly with hypokalemia (56%), hypocalcemia (44%), and hyponatremia (32%). Among these, hypokalemia (odds ratio: 2.43) and hypernatremia (odds ratio: 2.26) were significantly associated with mortality.

**Conclusion:**

Electrolyte abnormalities were highly prevalent among children in the pediatric intensive care unit, with imbalances in the calcium, potassium, and sodium levels being the most common. Hypokalemia and hypernatremia were significantly and positively associated with mortality.

## Introduction

Fluid and electrolyte balance are the key to maintaining physiological homeostasis and cellular metabolism.^1,2^ Electrolyte abnormalities in serum disrupt cellular functions, tissue perfusion, neural transmissions, muscle contractions, and acid–base balance.^1,2^ Managing serum electrolyte abnormalities is challenging for pediatricians because such disturbances manifest into a mild clinical condition and even lead to life-threatening events that necessitate admissions to intensive care units.^3–7^ Patients admitted to pediatric intensive care units (PICUs) are at a high risk of electrolyte disturbances due to their higher body water content, immature renal function, and clinical conditions, including dehydration, sepsis, renal failure, and gastrointestinal losses, among other factors.^7^ Specifically, in children admitted to PICUs, serum electrolyte abnormalities are common and associated with high rates of morbidity and mortality.^4,6,8^ Dysnatremia is commonly observed among patients in PICUs, with hyponatremia being the most common and recorded in approximately 40% of children.^7^ Hyponatremia and hypernatremia are predictors of poor clinical outcomes.^7,9^ Dyskalemia, particularly hypokalemia, is also a common serum electrolyte abnormality and is reported in approximately 22%–24% of patients admitted to PICUs.^3,7,10^ Hyperkalemia induces potentially lethal arrhythmias, cardiac dysfunction, among other complications, and is thus associated with a high mortality risk.^3^ Similarly, hypocalcemia is observed in around 20% children and is associated with higher mortality rates.^11^

In patients admitted to PICUs with imbalances in serum sodium, potassium, calcium, and phosphorus levels, early identification and appropriate interventions are crucial.^2,7^ In most resource-rich settings, electrolyte levels in children with critical illnesses are monitored routinely, which allows appropriate and timely interventions to manage electrolyte imbalances.

However, in resource-poor settings, such as the setting of the present study, regular monitoring of electrolyte abnormalities is rarely performed. In addition, limited studies have been conducted on electrolyte abnormalities among patients in PICUs in India.^5,12–18^ Some electrolyte abnormalities typically seen in resource-poor settings are associated with the type of patients seen in these settings—like children with severe acute malnutrition present with total body potassium deficiency.^12,13^ Children with tuberculous meningitis might have hypochloremic-hyponatremia due to cerebral salt wasting.^18^ Therefore, in the present study, we determined the prevalence of various electrolyte abnormalities and examined their associations with sociodemographic factors, clinical features, primary organ systems involved, and patient outcomes in the PICU of a tertiary care hospital in Ujjain, Madhya Pradesh. Understanding the factors contributing to electrolyte imbalances in this context can be valuable for improving clinical management and patient outcomes.

## Methods

### Study design and setting

This prospective observational study was conducted in the PICU of R.D. Gardi Medical College, Ujjain, Madhya Pradesh, for a 16-month period from January 1, 2023 to April 30, 2024. R.D. Gardi Medical College is located in a rural part of Ujjain district in central India. Madhya Pradesh is India’s second-largest state by area, with 72 million inhabitants and about 72% of the population residing in rural areas. The state predominantly comprises an agrarian population, with 35% vulnerable population having very low land holdings.^19^ In addition, the state is lagging in health indicators, with the highest infant mortality rate (IMR) of 40/1000 live births compared with India’s national average of 26/1000 live births.^20^ Rural areas of the state report an IMR of 43, higher than that of urban areas that have an IMR of 28/1000 live births—representing 1.5 times higher risk of death before 1 year of age.^20^

### Study population

Children aged between 1 month and 18 months admitted to the PICU for at least 24 h were considered eligible and included in the study. Patients leaving against medical advice or those who expired within the first 24 h of admission were excluded.

### Sample size estimation

The sample size was calculated based on an estimated 30%–50% prevalence of electrolyte abnormalities in critically ill children.^6^ Using a conservative estimate of 40% prevalence, with a 95% confidence interval and 5% absolute precision, the minimum sample size was estimated to be 369, which increased to 410 after adjusting for a 10% potential refusal rate.

### Data collection

Data were collected prospectively using a structured case record form. Demographic variables included age, sex, and socioeconomic status. Clinical data comprised the presenting complaints of the patients. Nutritional status was assessed using World Health Organization criteria for moderate and severe acute malnutrition (SAM).^21^ Pediatric sepsis was defined as systemic inflammatory response syndrome (SIRS) in the presence of suspected or proven infection (bacterial, viral, fungal, or parasitic) according to Goldstein et al. criteria.^22^ The data collection form is attached as Supplemental File 1. Primary outcomes were as follows: (a) serum electrolyte abnormalities, which were defined based on a literature review. The values defined for various electrolyte abnormalities are presented in Table 2; (b) association of serum electrolyte abnormalities with primary diagnoses classified according to the organ system involved; and (c) association of serum electrolyte abnormalities with mortality.

### Laboratory methods

All children included in the study underwent venous blood sampling (4 ml), which was collected in plain vacutainer tubes containing serum separator gel. After centrifugation at 3000 rpm for 5 min, serum was analyzed in the Central Clinical Biochemistry Laboratory located in hospital premises using the Vitros 250 Chemistry System (Ortho Clinical Diagnostics, Bengaluru, India). The electrolytes assessed included sodium, potassium, calcium, magnesium, and phosphorus. The electrolyte abnormalities presented in the study are based on the initial blood sample taken within first 2h after admission. Additional subsequent sampling was done based on clinical condition and electrolyte abnormalities, but is not presented here.

### Data management and statistical analysis

The data were entered in EpiData Entry (version 3.1) and then transferred to Stata 12.1 (Stata Corp., College Station, TX, USA) software for statistical analysis. Frequency and percentages are presented for categorical data. Descriptive statistics were used to summarize demographic and clinical characteristics. The frequency and types of electrolyte abnormalities were noted. Comparisons between categorical variables were made using the Chi-square or Fisher’s exact test, while continuous variables were analyzed using the student’s *t*-test or Mann–Whitney *U* test, as appropriate. Pearson Chi-square test was used to test for association of organ system -neurological, respiratory, sepsis, gastrointestinal, endocrine, musculoskeletal, hematology, renal and cardiovascular with each electrolyte abnormalities as outcome variables. Crude odds ratios (cOR) were reported for each of the electrolyte imbalances. Different stepwise multivariate logistic regression models with backward elimination were developed with each of the common electrolyte imbalances as the outcome variable. In bivariate analyses, a *p*-value of <0.1 was considered significant for entry into the model. The analysis was started with the full model, then the variable with highest *p*-value was removed, and model was revised and refitted with remaining predictors. The procedure was repeated till the *p*-value of all the predictor variables was less than 0.1 except age and sex; we designated this as final model. Adjusted ORs and their respective 95% confidence intervals (CIs) were then calculated from the final model. For the final model, model discrimination was done using C-statistics-receiver-operating-characteristics (ROC) curve, and goodness-of-fit test using the Pearson Chi-square test for each of the electrolyte abnormalities. Associations between electrolyte abnormalities and mortality were assessed using ORs with 95% CIs. A *p*-value of less than 0.05 was considered statistically significant.

### Ethical considerations

The Institutional Ethical Committee approved the study (Approval number IEC Ref No PG/Pediatrics/61/2022). The procedures followed were in accordance with the ethical standards set by the institutional ethics committee and with the Declaration of Helsinki. Prior to participation, written informed consent was obtained from the parents or Legally Authorized Representative of all minors. Assent was also obtained from all participants, including the minors themselves, in age-appropriate language. Participation was entirely voluntary, and participants were informed that they could withdraw at any time without affecting their care.

## Results

**Figure 1.**
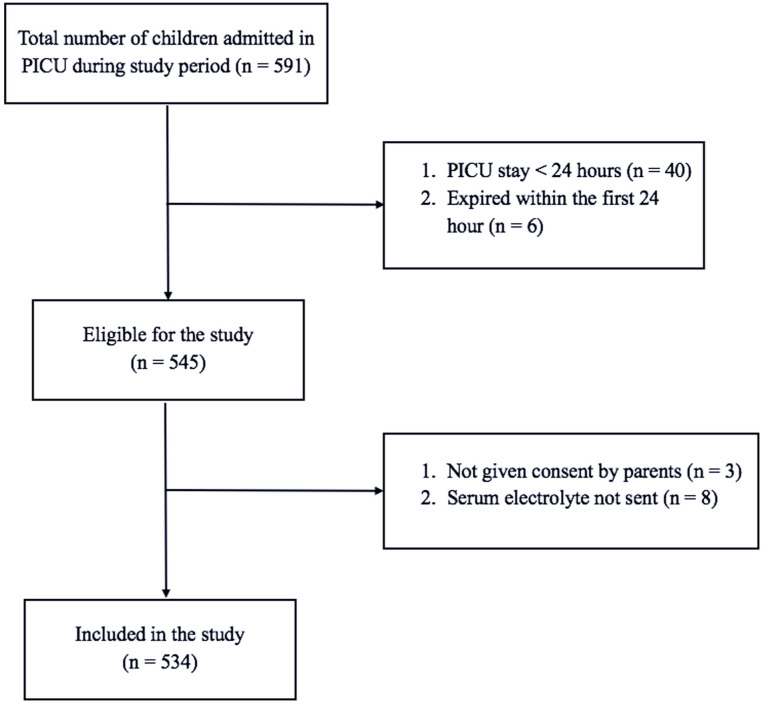
Flow chart of recruitment of study participants.

During the study period, a total of 534 patients aged between 1 month and 18 years were enrolled. Figure 1 shows the flow chart of patient enrollment. The mean (±SD) age of the children included was 5.13 (±4.79) years. Most patients (*n* = 317, 59%) were aged between 1 month and 5 years, followed by those aged more than 10 years (*n* = 122, 23%) and between 5 and 10 years (*n* = 95, 18%). Boys predominated the study population (*n* = 325, 61%), with girls constituting the remaining 39% (*n* = 209). A significant proportion of patients (449, 84%) belonged to the rural areas. The clinical features at admission classified according to the organ system involved are presented in Table 1. The central nervous system was involved in most cases (75%), followed by the respiratory system (66%) and gastrointestinal system (56%). A total of 34 (6%) patients were diagnosed as having severe SAM, and 68 (12%) patients had moderate SAM.

**Table 1.** Prevalence of clinical features classified according to the organ system involved among children admitted to the pediatric intensive care unit (*n* = 534).

Variable	*n* = 534 (%)
Neurological system	402 (75)
Irritability	111 (21)
Headache	88 (16)
Seizures	80 (15)
Altered consciousness	67 (13)
Hypotonia	33 (6)
Fasciculations	12 (3)
Tremors	6 (2)
Respiratory system	352 (66)
Respiratory distress	323 (60)
Gastrointestinal system	298 (56)
Loose stools	147 (28)
Vomiting/Nausea	105 (20)
Abdominal distention	46 (9)
Sepsis	285 (53)
Musculoskeletal system	180 (34)
Muscular pain	126 (24)
Joint pain/Arthralgia	54 (10)
Hematological system	173 (32)
Renal system	140 (26)
Oliguria	76 (14)
Hematuria	64 (12)
Cardiovascular system	59 (11)
Endocrine system	54 (10)
Polyuria	28 (5)
Polydipsia	26 (5)

In total, 1057 episodes of single electrolyte imbalances were observed in 534 patients. Table 2 shows the prevalence and severity of various electrolyte abnormalities in order of their frequency. Hypocalcemia was the most common electrolyte abnormality (*n* = 263, 25%), followed by hypokalemia (*n* = 192, 18%) and hyponatremia (*n* = 174, 16%). Among the 263 episodes of hypocalcemia, 173 (65%) were classified as severe. Twelve (6%) of the 192 episodes of hypokalemia were classified as critical or life-threatening. Around 80% (*n* = 27) of the patients with severe SAM (*n* = 34) had hypokalemia. Hyponatremia was observed in 174 episodes (16%), of which 162 (94%) were mild and 12 (6%) were severe. Patients with loose stools or diarrhea mostly experienced hyponatremia, with 147 such patients exhibiting 50 episodes (Table 1).

**Table 2.** The prevalence and severity of various electrolyte abnormalities in order of their frequency recorded in PICU in study population (*n* = 534).

Electrolyte imbalance (*n* = 1057)	Number (%)
Hypocalcemia (*n* = 263)	
Severe Hypocalcemia (<6.5 mg/dL)^21^	173 (66)
Hypokalemia (*n* = 192)	
Critical/Life Threatening (<2.5 mEq/L)^7^	12 (6)
Hyponatremia (*n* = 174)	
Severe Hyponatremia (<125 mEq/L)^7^	12 (7)
Hyperphosphatemia (*n* = 115)	
Severe Hyperphosphatemia (>6.8 mg/dL)^7^	17 (15)
Hypophosphatemia (*n* = 87)	
Severe Hypophosphatemia (<1.0 mg/dL)^7^	10 (11)
Hypernatremia (*n* = 60)	
Severe Hypernatremia (>150 mEq/L)^7^	21 (35)
Hypermagnesemia (*n* = 54)	
Severe Hypermagnesemia (>6.0 mg/dL)^22^	5 (9)
Hyperkalemia (*n* = 42)	
Severe Hyperkalemia (>7.0 mEq/L)^7^	7 (16)
Hypercalcemia (*n* = 41)	
Severe Hypercalcemia (>15.0 mg/dL)^23^	3 (7)
Hypomagnesemia (*n* = 29)	
Severe Hypomagnesemia (<1.0 mEq/L)^24^	5 (17)
**Combination of electrolyte imbalance**	**Number**
Hyponatremia and Hypokalemia	83
Hyperphosphatemia and Hyponatremia	53
Hypophosphatemia and Hyponatremia	45
Hyperphosphatemia and Hypokalemia	39
Hypophosphatemia and Hypokalemia	33
Hypokalemia and Hypocalcemia	32

Hyperphosphatemia occurred in 115 (11%) episodes, of which 98 (85%) were mild and 17 (15%) were severe. The central nervous system was the most common organ system involved, with tetany and muscle cramps as the main symptoms (*n* = 75, 65%). This was followed by the renal system (*n* = 40, 35%), with acute kidney injury as the primary diagnosis. Hypophosphatemia was observed in 87 (8%) episodes, with rickets (*n* = 67, 77%) being the most common diagnosis. A total of 20 episodes of hypophosphatemia were noted in 34 patients with SAM.

Among the 54 episodes of hypermagnesemia, 38 were observed in patients with cardiovascular disease symptoms. Similarly, 16 of the 29 episodes of hypomagnesemia were observed in children with SAM.

Incidence of more than one electrolyte abnormality was also observed in the study population. Specifically, 67 episodes of the three common electrolyte abnormalities—hypocalcemia, hypokalemia, and hyponatremia—were recorded. The combination hypocalcemia–hyponatremia was found to be the most common, with 128 episodes, followed by the combination hypocalcemia–hypokalemia, with 112 episodes.

Table 3 shows the association of four common electrolyte imbalances with the different organ systems. Among the 1057 episodes of electrolyte abnormalities, 263 were related to hypocalcemia, with 173 (16%) cases being severe (<6.5 mg/dL). All children with hypocalcemia had neurological involvement. Hypocalcemia was significantly associated with the endocrine system (crude OR 2.12, 95% CI 1.47–3.07, *p* < 0.001), musculoskeletal system (crude OR 1.75, 95% CI 1.21–2.52, *p* = 0.003), renal system (crude OR 2.03, 95% CI 1.36–3.01, *p* < 0.001), and cardiovascular system (crude OR 2.18, 95% CI 1.23–3.85, *p* = 0.007). However, none of the above systems remained significant on multiple stepwise logistic regression. Hypokalemia was significantly associated with the neurological system (adjusted OR 2.07, 95% CI 1.23–3.49, *p* = 0.006), gastrointestinal system (adjusted OR 2.23, 95% CI 1.50–3.32, *p* < 0.001), endocrine system (adjusted OR 2.26, 95% CI 1.49–3.43, *p* < 0.001), and cardiovascular system (adjusted OR 10.20, 95% CI 4.96–20.99, *p* < 0.001). Hyponatremia was significantly associated with the respiratory system (adjusted OR 2.82, 95% CI 1.79–4.44, *p* < 0.001) and the gastrointestinal system (adjusted OR 1.95, 95% CI 1.29–2.94, *p* = 0.001). Hyperkalemia was significantly associated with the neurological system (adjusted OR 3.84, 95% CI 1.85–7.97, *p* < 0.001), endocrine system (adjusted OR 2.24, 95% CI 1.13–4.44, *p* = 0.020), and cardiovascular system (adjusted OR 3.53, 95% CI 1.54–8.10, *p* = 0.003). The models for the remaining electrolyte disturbances could not be fitted due to an inadequate number of observations in the rows or columns.

**Table 3.** Association of electrolyte imbalances with organ system involvement: prevalence, crude, and adjusted ORs (*n* = 534).

Involved organ system	Hypocalcemia *n* (%)		Crude OR (95% CI)	*p*-Value	Adjusted OR (95% CI)	*p*-Value
	Yes (*n* = 263)	No (*n* = 271)				
Neurological (*n* = 407)						
No	0 (0)	127 (47)	R	R	—	—
Yes	263 (100)	144 (53)	—	—	—	—
Respiratory (*n* = 323)						
No	99 (38)	112 (41)	R	R	—	—
Yes	164 (62)	159 (59)	1.16 (0.82–1.65)	0.384	—	—
Sepsis (*n* = 285)						
No	127 (48)	122 (45)	R	R	-	-
Yes	136 (52)	149 (55)	0.87 (0.62–1.23)	0.449	-	-
Gastrointestinal (*n* = 227)						
No	143 (54)	164 (61)	R	R	—	—
Yes	120 (46)	107 (39)	1.28 (0.91–1.81)	**0.151**	—	—
Endocrine (*n* = 182)						
No	151 (57)	201 (74)	R	R	—	—
Yes	112 (43)	70 (26)	2.12 (1.47–3.07)	**0.000**	—	—
Musculoskeletal (*n* = 174)						
No	161 (61)	199 (73)	R	R	—	—
Yes	102 (39)	72 (27)	1.75 (1.21–2.52)	**0.003**	—	—
Hematologic (*n* = 173)						
No	175 (67)	186 (69)	R	R	—	—
Yes	88 (33)	85 (31)	1.10 (0.76–1.58)	0.605	—	—
Renal (*n* = 140)						
No	176 (67)	218 (80)	R	R	—	—
Yes	87 (33)	53 (20)	2.03 (1.36–3.01)	**0.000**	—	—
Cardiovascular (*n* = 59)						
No	224 (85)	251 (93)	R	R	—	—
Yes	39 (15)	20 (7)	2.18 (1.23–3.85)	**0.007**	—	—
Hypokalemia						
Involved organ system	Hypokalemia *n* (%)		Crude OR (95% CI)	*p*-Value	Adjusted OR (95% CI)	*p*-Value
	Yes (*n* = 192)	No (*n* = 342)				
Neurological (*n* = 407)						
No	24 (12)	103 (30)	R	R	R	R
Yes	168 (88)	239 (70)	3.01 (1.85–4.90)	**0.000**	2.07 (1.23–3.49)	**0.006**
Respiratory (*n* = 323)						
No	79 (41)	132 (39)	R	R	—	—
Yes	113 (59)	210 (61)	0.89 (0.62–1.28)	0.563	—	—
Sepsis (*n* = 285)						
No	84 (44)	165 (48)	R	R	—	—
Yes	108 (56)	177 (52)	1.19 (0.84–1.70)	0.318	—	—
Gastrointestinal (*n* = 227)						
No	86 (45)	221 (65)	R	R	R	R
Yes	106 (55)	121 (35)	2.25 (1.56–3.22)	**0.000**	2.23 (1.50–3.32)	**0.000**
Endocrine (*n* = 182)						
No	103 (54)	249 (73)	R	R	R	R
Yes	89 (46)	93 (27)	2.31 (1.59–3.35)	**0.000**	2.26 (1.49–3.43)	**0.000**
Musculoskeletal (*n* = 174)						
No	119 (62)	241 (70)	R	R	—	—
Yes	73 (38)	101 (30)	1.46 (1.00–2.12)	**0.045**	—	—
Hypokalemia						
Involved organ system	Hypokalemia *n* (%)		Crude OR (95% CI)	*p*-Value	Adjusted OR (95% CI)	*p*-Value
	Yes (*n* = 192)	No (*n* = 342)				
Hematological (*n* = 173)						
No	124 (65)	237 (69)	R	R	—	—
Yes	68 (35)	105 (71)	1.23 (0.85–1.80)	0.264	—	—
Renal (*n* = 140)						
No	124 (65)	270 (79)	R	R	—	—
Yes	68 (35)	72 (21)	2.05 (1.38–3.04)	**0.000**	—	—
Cardiovascular (*n* = 59)						
No	144 (75)	331 (97)	R	R	R	R
Yes	48 (25)	11 (3)	10.03 (5.06 -19.87)	**0.000**	10.20 (4.96–20.99)	**0.000**
Hyponatremia						
Involved organ system	Hyponatremia *n* (%)		Crude OR (95% CI)	*p*-Value	Adjusted OR (95% CI)	*p*-Value
	Yes (*n* = 174)	No (*n* = 360)				
Neurological (*n* = 407)						
No	5 (3)	127 (35)	R	R	—	—
Yes	169 (97)	233 (65)	1.82	0.48	—	—
Respiratory (*n* = 323)						
No	38 (22)	173 (48)	R	R	R	R
Yes	136 (78)	187 (52)	3.31 (2.18–5.01)	**0.000**	2.82 (1.79–4.44)	**0.000**
Sepsis (*n* = 285)						
No	85 (49)	164 (46)	R	R	—	—
Yes	89 (51)	196 (54)	0.87 (0.60–1.25)	0.474	—	—
Gastrointestinal (*n* = 227)						
No	81 (47)	226 (63)	R	R	R	R
Yes	93 (53)	134 (37)	1.93 (1.34–2.79)	**0.000**	1.95 (1.29–2.94)	**0.001**
Endocrine (*n* = 182)						
No	100 (57)	252 (70)	R	R	—	—
Yes	74 (43)	108 (30)	1.72 (1.18–2.51)	**0.004**	—	—
Musculoskeletal (*n* = 174)						
No	106 (61)	254 (71)	R	R	—	—
Yes	68 (39)	106 (29)	1.53 (1.05–2.24)	**0.026**	—	—
Hematological (*n* = 173)						
No	121 (70)	240 (67)	R	R	—	—
Yes	53 (30)	120 (33)	0.87 (0.59–1.29)	0.506	—	—
Renal (*n* = 140)						
No	121 (70)	273 (76)	R	R	—	—
Yes	53 (30)	87 (24)	1.37 (0.91–2.05)	**0.122**	—	—
Cardiovascular (*n* = 59)						
No	148 (85)	327 (91)	R	R	—	—
Yes	26 (15)	33 (9)	1.74 (1.00–3.01)	**0.048**	—	—
Hyperkalemia						
Involved organ system	Hyperkalemia *n* (%)		Crude OR (95% CI)	*p*-Value	Adjusted OR (95% CI)	*p*-Value
	Yes (*n* = 42)	No (*n* = 492)				
Neurological (*n* = 407)						
No	25 (60)	382 (78)	R	R	R	R
Yes	17 (40)	110 (22)	2.36 (1.23–4.53)	**0.010**	3.84 (1.85–7.97)	**0.000**
Respiratory (*n* = 323)						
No	18 (43)	193 (39)	R	R	—	—
Yes	24 (57)	299 (61)	0.86 (0.45–1.62)	0.644	—	—
Sepsis (*n* = 285)						
No	21 (50)	228 (46)	R	R	—	—
Yes	21 (50)	264 (54)	0.86 (0.45–1.62)	0.648	—	—
Gastrointestinal (*n* = 227)						
No	23 (55)	284 (58)	R	R	—	—
Yes	19 (45)	208 (42)	1.12 (0.59–2.12)	0.710	—	—
Endocrine (*n* = 182)						
No	22 (52)	330 (67)	R	R	R	R
Yes	20 (48)	162 (33)	1.85 (0.98–3.49)	**0.057**	2.24 (1.13–4.44)	**0.020**
Musculoskeletal (*n* = 174)						
No	34 (81)	326 (66)	R	R	—	—
Yes	8 (19)	166 (34)	0.46 (0.20–1.02)	**0.056**	—	—
Hematological (*n* = 173)						
No	33 (79)	328 (67)	R	R	—	—
Yes	9 (21)	164 (33)	0.54 (0.25–1.16)	**0.118**	—	—
Renal (*n* = 140)						
No	29 (69)	365 (74)	R	R	—	—
Yes	13 (31)	127 (26)	1.28 (0.64–2.55)	0.468	—	—
Cardiovascular (*n* = 59)						
No	32 (76)	443 (90)	R	R	R	R
Yes	10 (24)	49 (10)	2.82 (1.30–6.09)	**0.008**	3.53 (1.54–8.10)	**0.003**

Statistically significant values are highlighted in bold.

### AUROC and goodness-of-fit for electrolyte abnormalities

The AUROC for regression model for hypokalemia was 75.60 and goodness-of-fit *p*-value was 0.065. The AUROC for regression model for hyponatremia was 66.26 and goodness-of-fit *p*-value was 0.361. The AUROC for regression model for hyperkalemia was 69.03 and goodness-of-fit *p*-value was 0.042.

Table 4 shows the association of single electrolyte imbalance with mortality. Hypokalemia (OR = 2.39, 95% CI 1.18–4.82; *p* = 0.015) and hypernatremia (OR = 2.65, 95% CI 1.14–6.15; *p* = 0.023) exhibited statistically significant associations with mortality. Combination of electrolyte abnormalities was also frequently associated with mortality. Specifically, the combination of hypocalcemia with hypokalemia contributed to the maximum number of deaths (*n* = 12, 35%), followed by the combinations of hyponatremia with hypocalcemia (7 deaths, 21%) and hyponatremia with hypokalemia (7 deaths, 21%). Mortality rates were the highest among patients with neurological system involvement (*n* = 25, 89%), followed by those with respiratory system involvement (24 deaths, 70%) and gastrointestinal system involvement (15 deaths, 44%).

**Table 4.** Association of electrolyte imbalance with mortality (*n* = 34 deaths).

Electrolyte imbalance	Mortality (*n* = 34, %)	OR	95% CI	*p*-Value
Hypokalemia	19 (56)	2.39	1.18–4.82	0.015
Hypocalcemia	15 (44)	0.80	0.39–1.61	0.537
Hyponatremia	11 (32)	0.98	0.47–2.07	0.976
Hypophosphatemia	9 (26)	1.94	0.87–4.33	0.102
Hypernatremia	8 (24)	2.65	1.14–6.15	0.023
Hyperphosphatemia	6 (18)	0.76	0.31–1.90	0.570
Hypercalcemia	2 (6)	0.73	0.17–3.19	0.686
Hypermagnesemia	1 (3)	0.25	0.03–1.90	0.183
Hyperkalemia	1 (3)	0.33	0.45–2.54	0.293
**Combination of electrolytes**				
Hyponatremia with hypokalemia	5 (15)	0.93	0.35–2.48	0.889
Hyperphosphatemia with hyponatremia	3 (9)	0.87	0.25–2.95	0.824
Hypophosphatemia with hyponatremia	3 (9)	1.05	0.30–3.59	0.931
Hyperphosphatemia with hypokalemia	1 (3)	0.36	0.04–2.76	0.332
Hypophosphatemia with hypokalemia	5 (15)	2.90	1.04–8.08	0.041
Hypokalemia with hypocalcemia	5 (15)	3.02	1.08–8.42	0.035

CI: confidence intervals; OR: odds ratio.

## Discussion

We observed 1057 episodes of single electrolyte imbalance in the study population, with hypocalcemia (25%), hypokalemia (18%), and hyponatremia (16%) being the common disorders.

Demographically, the study population showed a male predominance (61%), and most of the patients were from rural areas (84%), with 59% of children under 5 years of age. These characteristics suggest that female children residing in rural settings may be more vulnerable to severe illnesses at admission, potentially due to delayed healthcare access and/or nutritional deficiencies.^25,26^ Since the last two decades, gender differences in healthcare seeking, diet, and immunization coverage have been highly prevalent in India.^25,26^ The high prevalence of malnutrition (6% severe, 12% moderate) would have further exacerbated electrolyte imbalances, particularly hyponatremia and hypokalemia.^27^ In the present study, approximately 80% (*n* = 27) of patients with severe SAM (*n* = 34) had hypokalemia. This finding is consistent with that of a study conducted in children with SAM in Pakistan.^27^

Majority of the patients (*n* = 402, 75%) were admitted with neurological symptoms (Table 2). All children with hypocalcemia had neurological involvement. Hypocalcemia was also significantly associated in bivariate analysis with the endocrine system (crude OR 2.12, *p* < 0.001), musculoskeletal system (crude OR 1.75, *p* = 0.003), renal system (crude OR 2.03, *p* < 0.001), and cardiovascular system (crude OR 2.18, *p* = 0.007). These findings align with those of a study involving 38 children with hypocalcemia, with 49% displaying neurological signs and 42% exhibiting cardiovascular abnormalities in the form of electrocardiogram changes.^11^ Hypocalcemia was reported as the most frequent serum electrolyte abnormality in another study from Pakistan.^4^ However, the study reported a prevalence of 57.6%, which is much higher than that in the present study.^4^ This discrepancy might be due to population differences between the two settings. The association with neurological symptoms, such as seizures, underscores the need for vigilant monitoring of calcium levels in patients admitted to PICUs to prevent complications such as seizures or arrhythmias.^28^ However, we did not measure ionized calcium in our study. Total calcium should be used only as a screening tool and ionized calcium should be used for calcium homeostasis, especially in critically ill and in children with albumin abnormalities.^28^

Hypokalemia, with 192 (18%) episodes and 12 severe episodes (serum potassium < 2.5 mEq/L), showed a statistically significant association with mortality (OR: 2.39; 95% CI: 1.18–4.82; *p* = 0.015). A much higher prevalence of 40% was reported in a study from Boston, USA,^10^ which documented a direct relation between the severity of hypokalemia and mortality (OR = 2.2, *p* = 0.003), consistent with the findings of the present study. Studies have reported hypokalemia as the most common electrolyte disorder in children above 4 years of age.^6,7^ Notably, 75% of patients with cardiovascular system involvement exhibited hypokalemia with risk for cardiovascular system involvement in patients with hypokalemia was significantly high (adjusted OR 10.20; *p* < 0.001), likely due to its impact on cardiac repolarization and muscle function.^3,29^ Furthermore, we identified gastrointestinal losses (adjusted OR 2.23; *p* < 0.001) and the endocrine system (adjusted OR 2.26; *p* < 0.001) as significant risk factors for hypokalemia in our patients, consistent with mechanisms such as vomiting and diuretic use in children with critical illnesses.^3,29^ The most common endocrine mechanism for hypokalemia is increased renal potassium excretion due to mineralocorticoid excess or increased aldosterone effect.^7^

In our study hyponatremia occurred in 174 (16%) episodes, and all episodes were associated with neurological system and were significantly associated with respiratory system (adjusted OR 2.82, 95% CI 1.79–4.44, *p* < 0.001) and gastrointestinal system (adjusted OR 1.95, 95% CI 1.29–2.94, *p* = 0.001). Syndrome of Inappropriate Antidiuretic Hormone Secretion (SIADH) and cerebral salt wasting are common mechanisms of hyponatremia in neurological diseases.^7^ A study of community-acquired infections reported 53% prevalence of hyponatremia in a cohort of 400 patients.^30^ The etiologies reported in the study were gastroenteritis (43%), bronchiolitis (57%), and pyelonephritis (68%),^30^ aligning with the findings of the current study. Hyponatremia has been reported to be the most common electrolyte abnormality, particularly in children aged less than 4 years.^6,7^ Severe hyponatremia, defined as serum sodium levels of <125 mEq/L, was less common (1%) but contributed to mortality in combination with other electrolyte imbalances. The multifactorial etiology, including fluid losses and dilutional effects, highlights the complexity of sodium management in PICU settings.^7,9,31^

Hyperkalemia was significantly associated with risk for neurological system (adjusted OR 3.84, *p* < 0.001) involvement, endocrine system (adjusted OR 2.24, *p* = 0.020), and cardiovascular system (adjusted OR 3.53, *p* = 0.003) (Table 3). Hyperkalemia reduces the K⁺ gradient leading to depolarization of the neural membranes, which initially increases excitability but later causes conduction block and can lead to muscle weakness, paralysis, and sensory disturbances.^3,29^ Hyperkalemia is associated with endocrine disorders mainly due to aldosterone deficiency or resistance, as in Addison’s disease.^3,29^ Low aldosterone reduces potassium excretion by the kidneys, causing hyperkalemia. In addition, insulin deficiency (like in diabetic ketoacidosis) causes potassium to shift from cells into the blood, raising serum potassium levels.^3,29^ Severe hypothyroidism can also mildly increase potassium by reducing cellular uptake.^3,29^ Hyperkalemia affects the heart by reducing the resting membrane potential, which slows electrical conduction leading to typical ECG changes and varied symptoms like palpitations, bradycardia, hypotension, and possibly cardiac arrest if untreated.^3,29^

Hypernatremia, though less frequent (6%), showed a significant association with mortality (OR: 2.65; 95% CI: 1.14–6.15; *p* = 0.023), particularly in patients with neurological system involvement (3% severe cases). This finding suggests that severe hypernatremia (serum sodium >150 mEq/L) might have been rapidly corrected leading to cerebral edema.^3,7^ Hypernatremia complicates dehydration by causing cellular dehydration, especially in brain, leading to neurological symptoms. It can also lead to intracranial hemorrhage due to brain shrinkage and can worsen circulatory dysfunction and cause renal dysfunction.^3,7^

The lower prevalence of hyperphosphatemia (11%), hypermagnesemia (5%), hyperkalemia (4%), and hypomagnesemia (3%) indicates their less profound but clinically significant impact, particularly in patients with renal or musculoskeletal involvement.^7,24,32^ In the present study, patients with SAM had hypokalemia, hypocalcemia, hypophosphatemia, and hypomagnesemia. A study from Pakistan reported the prevalence of hypokalemia, hypocalcemia, hyponatremia, and hypomagnesemia as 80%, 72%, 14%, and 49%, respectively, among 184 children with SAM.^27^

In addition to the contribution of the hypokalemia–hypernatremia combination to mortality, the joint prevalence of hypocalcemia and hypokalemia was associated with 35% of the deaths. Involvement of the neurological system was the most significant risk factor for mortality (89%), followed by the respiratory (70%) and gastrointestinal (44%) systems, reflecting the systemic impact of electrolyte disturbances.^7^ The higher mortality rate in patients with neurological involvement may be linked to the propensity of electrolyte imbalances to cause seizures and/or altered sensorium.^3,7^ Hypophosphatemia with hypokalemia was statistically significantly associated with mortality (OR 2.90, 95% CI 1.04–8.08; *p* = 0.041). The important causes were SAM, diarrhea, renal tubular acidosis and sepsis.^10^ Hypokalemia with hypocalcemia was also statistically significantly associated with mortality (OR 3.02, 95% CI 1.08–8.42; *p* = 0.035). Underlying hypomagnesemia was the most significant cause of this combination of electrolyte imbalance, with other causes being diarrhea or gastrointestinal causes, followed by renal tubular acidosis.^7,10^

Children who expired within 24 h of admission were excluded from the present study. This was done to minimize biases related to peripartum physiological instability, where electrolyte levels may not truly reflect underlying disease processes but rather terminal events. Including such cases could have led to overestimation of electrolyte abnormalities and confounding by acute resuscitation measures such as fluid therapy or inotropes. Moreover, clinical outcomes and associations are difficult to interpret as mortality was more likely related to the severity of primary illness rather than electrolyte imbalances per se. Excluding these children ensure greater homogeneity of the study population and improve the validity of observed associations.

### Limitations of the study

Our study has limitations—we did not monitor severity of disease using the Pediatric Risk of Mortality (PRISM) score system. However, PRISM III needs data collection for 12–24 h. This long period of time reflects not only the initial severity but also the management during this period.^33^ Also, the coefficients of each variable necessary to calculate the probability of death are not in the public domain.^33^ Although serial monitoring of serum electrolytes was done, only the initial electrolyte imbalances are presented in the study. Although our study reports an association between hypokalemia and hypernatremia with mortality the CIs around the odds ratios are quite wide for both the associations (Table 4). This might be due to lower number of deaths (*n* = 34) during the study period. In view of the observational study design, no causal associations can be drawn. Use of ionized calcium and ionized magnesium could have further strengthened the study.

## Conclusions

This study found a high prevalence of electrolyte imbalances among children admitted to the PICU. Hypocalcemia, hypokalemia, and hyponatremia were identified as the common disorders in the study cohort. We also analyzed the correlations between electrolyte abnormalities and various clinical features involving different organ systems. Organ system involvement differed with electrolyte imbalance. Our findings highlight the notable impact of electrolyte imbalances on patient outcomes, with hypokalemia being most strongly related to mortality, followed by hypocalcemia and hypernatremia. Combinations of electrolyte abnormalities, such as hypophosphatemia with hypokalemia and hypokalemia with hypocalcemia, were also statistically significantly associated with mortality.

## Supplemental Material

sj-docx-1-smo-10.1177_20503121251391990 – Supplemental material for Prevalence of Electrolyte Abnormality and its Correlation with Clinical Features and Patient Outcomes in Children Admitted to Pediatric Intensive Care Unit of a Resource-Constrained Setting in India Supplemental material, sj-docx-1-smo-10.1177_20503121251391990 for Prevalence of Electrolyte Abnormality and its Correlation with Clinical Features and Patient Outcomes in Children Admitted to Pediatric Intensive Care Unit of a Resource-Constrained Setting in India by Arushi Gupta, Sunil Kasundriya, Shreya Shrivastava, Manju Purohit and Ashish Pathak in SAGE Open Medicine
