# Supplementary material for: Prevalence of Electrolyte Abnormality and its Correlation with Clinical Features and Patient Outcomes in Children Admitted to Pediatric Intensive Care Unit of a Resource-Constrained Setting in India
Source: SAGE Open Med. 2025 Nov 29;13:20503121251391990. doi: 10.1177/20503121251391990 (PMC12665032; doi:10.1177/20503121251391990)
Supplement: sj-docx-1-smo-10.1177_20503121251391990 – Supplemental material for Prevalence of Electrolyte Abnormality and its Correlation with Clinical Features and Patient Outcomes in Children Admitted to Pediatric Intensive Care Unit of a Resource-Constrained Setting in India [file sj-docx-1-smo-10.1177_20503121251391990.docx]

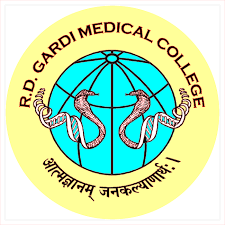
 **PICU study- Questionnaire**

**Prevalence of Electrolyte Abnormality and its Correlation with Clinical Features and Patient Outcomes in Children Admitted to Pediatric Intensive Care Unit of a Resource Constrained Setting in India**

**Patient Information:**

1. IPD No: ____________
2. Name: ______________________________
3. Age: ____________ month/year
4. Sex: ☐ Male ☐ Female
5. Date of Admission in PICU: (DD/MM/YYYY) ________________________
6. Date of Discharge: __________________________
7. Date of Death (If applicable): _________________
8. Duration of stay: ____________________________ (in days)

9. Locality: ☐ Urban ☐ Rural

**Disease-related information:**

10. Presenting complaints on admission:

| **Complaints** |  |  | **Days** | **Complaints** |  |  | **Days** |
| --- | --- | --- | --- | --- | --- | --- | --- |
| 1. Fever | Yes | No |  | 16. Polyuria | Yes | No |  |
| 2. Headache | Yes | No |  | 17. Polydipsia | Yes | No |  |
| 3. Altered consciousness | Yes | No |  | 18. Oliguria | Yes | No |  |
| 4. Irritability | Yes | No |  | 19. Hematuria | Yes | No |  |
| 5. Seizures | Yes | No |  | 20. Fatigue | Yes | No |  |
| 6. Tremors | Yes | No |  | 21. Malaise | Yes | No |  |
| 7. Hypotonia | Yes | No |  | 22. Joint pain/Arthralgia | Yes | No |  |
| 8. Fasciculations | Yes | No |  | 23. Muscular pain | Yes | No |  |
| 9. Fast breathing | Yes | No |  | 24. Short stature | Yes | No |  |
| 10. Respiratory distress | Yes | No |  | 25. Congestive  cardiac failure | Yes | No |  |
| 11. Bleeding disorder- Platelet disorder/Factor disorder | Yes | No |  | 26. Cyanosis | Yes | No |  |
| 12. Anaemia | Yes | No |  |  |  |  |  |
| 13. Vomiting/Nausea | Yes | No |  |  |  |  |  |
| 14. Loose stools | Yes | No |  |  |  |  |  |
| 15. Abdominal  Distention | Yes | No |  |  |  |  |  |

11. System Involvement:

- Neurological system: ☐ Yes ☐ No
  - Irritability ☐ Yes ☐ No
  - Headache ☐ Yes ☐ No
  - Seizures ☐ Yes ☐ No
  - Altered consciousness ☐ Yes ☐ No
  - Hypotonia ☐ Yes ☐ No
  - Fasciculations ☐ Yes ☐ No
  - Tremors ☐ Yes ☐ No
- Respiratory system: ☐ Yes ☐ No
  - Fast breathing ☐ Yes ☐ No
  - Respiratory distress ☐ Yes ☐ No
  - X-ray suggestive pneumonia ☐ Yes ☐ No
- Gastrointestinal system: ☐ Yes ☐ No
  - Diarrhoea ☐ Yes ☐ No

Acute ☐ Yes ☐ No Chronic ☐ Yes ☐ No

- Vomiting/Nausea ☐ Yes ☐ No
- Abdominal distention ☐ Yes ☐ No
- Jaundice ☐ Yes ☐ No
- Hepatomegaly ☐ Yes ☐ No
- Evidence of pancreatitis ☐ Yes ☐ No
- Celiac disease ☐ Yes ☐ No
- Sepsis (Goldstein definition et al., 2005) ☐ Yes ☐ No
- Musculoskeletal system: ☐ Yes ☐ No
  - Fatigue ☐ Yes ☐ No
  - Malaise ☐ Yes ☐ No
  - Joint pain/Arthralgia ☐ Yes ☐ No
  - Muscular pain ☐ Yes ☐ No
- Hematological system: ☐ Yes ☐ No
  - Anaemia – Nutritional/Hemolytic/Bone marrow failure ☐ Yes ☐ No
  - Bleeding disorder - Platelet disorder/Factor deficiency ☐ Yes ☐ No
- Renal system: ☐ Yes ☐ No
  - Oliguria ☐ Yes ☐ No
  - Hematuria ☐ Yes ☐ No
  - Nephrotic syndrome ☐ Yes ☐ No
  - Acute kidney disease ☐ Yes ☐ No
- Cardiovascular system: ☐ Yes ☐ No
  - Congestive Cardiac Failure ☐ Yes ☐ No
  - Congenital Heart Disease - Cyanotic/Non-cyanotic ☐ Yes ☐ No
  - Acquired Heart Diseases – Kawasaki disease/Rheumatic Heart Disease

☐ Yes ☐ No

- Endocrine system: ☐ Yes ☐ No
  - Short stature ☐ Yes ☐ No
  - Hypothyroidism ☐ Yes ☐ No
  - Hypopituitarism ☐ Yes ☐ No
- Miscellaneous: ☐ Yes ☐ No

(If yes, please specify)

__________________________________________________________

12. Full Diagnosis at discharge:

__________________________________________________________________

__________________________________________________________________

13. Nutritional status (As defined by WHO):

- - SAM: ☐ Yes ☐ No
  - MAM: ☐ Yes ☐ No
  - Normal: ☐ Yes ☐ No

14. Electrolyte levels

| **Date** | **On admission** | Follow-up date | Follow-up |
| --- | --- | --- | --- |
| Sodium (mmol/L) |  |  |  |
| Potassium (mmol/L) |  |  |  |
| Calcium (mg/dl) |  |  |  |
| Magnesium (mg/dl) |  |  |  |
| Phosphorus (mmol/L) |  |  |  |

15. Electrolyte imbalance details:

| **S.no** | **Electrolyte** | **Imbalance** | **Range** |
| --- | --- | --- | --- |
| 1 | Sodium | a) Hyponatremia: ☐ Yes ☐ No  Severe Hyponatremia (< 125 mEq/L) | 1-3 years: - 134-144 mmol/L  4-11 years: - 134-143 mmol/L  12-18 years: - 135-145 mmol/L |
|  |  | b) Hypernatremia: ☐ Yes ☐ No  Severe Hypernatremia (> 150 mEq/L) |  |
| 2 | Phosphorus | a) Hypophosphatemia: ☐ Yes ☐ No  Severe Hypophosphatemia (< 1.0 mg/dL) | 1month-5 years: - 9.8-8.2 mg /dl  1-3 years: - 3.8-6.5 mg/dl  4-11 years: - 3.7-5.6 mg/dl  12-15 years: - 2.9-5.4 mg/dl  16-19 years: - 2.7-4.7 mg/dl) |
|  |  | b) Hyperphosphatemia: ☐ Yes ☐ No  Severe Hyperphosphatemia (> 6.8 mg/dL) |  |
| 3 | Calcium | a) Hypocalcemia: ☐ Yes ☐ No  Severe Hypocalcemia (< 6.5 mg/dL) | 1month-11 years: - 3.8-10.8 mg/dl  12-18 year: - 8.4-10.2 mg/dl |
|  |  | b) Hypercalcemia: ☐ Yes ☐ No  Severe Hypercalcemia (> 15.0 mg/dL) |  |
| 4 | Magnesium | a) Hypomagnesemia: ☐ Yes ☐ No  Severe Hypomagnesemia (< 1.0 mEq/L) | 1month: - 2 years: - 1.6-2.6 mg/dl  2-14 years: - 1.5-2.3 mg/dl  Conversion factor: - 0.411 |
|  |  | b) Hypermagnesemia: ☐ Yes ☐ No  Severe Hypermagnesemia (> 6.0 mg/dL) |  |
| 5 | Potassium | a) Hypokalemia: ☐ Yes ☐ No  Critical/Life Threatening (< 2.5 mEq/L) | 1-6 month: - 3.5-5.6 mmol/L  6 month-1year: - 3.5-6.1 mmol/L  > 1 year: - 3.3-4.6 mmol/L |
|  |  | b) Hyperkalemia: ☐ Yes ☐ No  Severe Hyperkalemia (> 7.0 mEq/L) |  |

17. Patient status/outcome:

1) Discharge

2) Discharge on request

3) Leave against medical advice

4) Death
